# Editorial: Molecular characterization of thyroid lesions in the era of “next generation” techniques, volume III

**DOI:** 10.3389/fendo.2026.1926803

**Published:** 2026-07-20

**Authors:** Umberto Malapelle, Dario de Biase

**Affiliations:** 1 Department of Public Health, University Federico II of Naples, Naples, Italy; 2 Solid Tumor Molecular Pathology Laboratory, IRCCS Azienda Ospedaliero-Universitaria di Bologna, Bologna, Italy; 3 Department of Pharmacy and Biotechnology (FABIT), University of Bologna, Bologna, Italy

**Keywords:** biological markers, molecular pathology, preoperative evaluation of thyroid nodules, thyroid disease, thyroid tumors

The evolution of thyroid disease in the era of next-generation technologies has profoundly transformed the way we interpret, classify, and manage thyroid lesions. Until a few years ago, the characterization of thyroid tumors was based almost exclusively on morphological, immunohistochemical, and clinical-radiological parameters; today, the molecular profile represents an essential component of integrated diagnostics and precision medicine. This Research Topic collects nine contributions that, taken together, clearly outline the transition from a descriptive view of thyroid disease to a multilevel interpretative model, in which driver mutations, transcriptomic profiles, serological biomarkers, predictive algorithms, and new sequencing platforms converge towards a more accurate stratification of biological and clinical risk.

A first thematic axis that emerges strongly concerns the role of *BRAF* alterations, which have long been considered central to the biology of papillary thyroid cancer, but are here reinterpreted from a broader, more dynamic perspective. The case series by Yamin et al. documented the real-world use of combinations of BRAF and MEK inhibitors other than the classical dabrafenib-trametinib regimen in patients with BRAF V600E-mutated advanced thyroid cancer, including cases of papillary and anaplastic carcinoma. Even in an extremely limited cohort, the work convincingly shows that precision medicine does not end with the identification of the target mutation, but requires continuous therapeutic modulation in the light of toxicity, resistance, and sequential strategies. The message is relevant: molecular characterization enables therapy, but clinical practice highlights how much the management of advanced thyroid tumors requires flexibility, longitudinal monitoring, and contextual interpretation of the molecular data.

At the prognostic level, Liu and Wei investigated the interactions between *BRAF* and *TP53* in papillary thyroid carcinoma, proposing a more nuanced interpretation of the relationships among mutations, pathways, and clinical behavior. Their study suggests that *BRAF*, *TERT*, and *TP53* should not be considered only as isolated markers, but as nodes of a biological network capable of redefining conventional prognostic models. In particular, the integration of mutational profiling and pathway analysis reinforces the idea that the clinical value of sequencing lies not solely in the list of identified variants but in understanding the functional context in which these alterations act. In this sense, the work fits fully into the trajectory of precision medicine, shifting the focus from the single gene to the biological system.

Still on the *BRAF* side, but with an even more specific focus on a clinically relevant endpoint, Yang et al. developed a nomogram to predict the risk of lung metastases in patients with papillary thyroid cancer under 55 years of age. The most interesting aspect of the study is the integration of clinicopathologic features and molecular profiles, including BRAF p.Val600Glu (p.V600E) and *TERT* promoter mutations in the predictive model. The apparently counterintuitive finding that the BRAF p.V600E mutation was associated with a lower risk of lung metastases in the subgroup studied reminds us how much the clinical relevance of a mutation depends on the biological and clinical context in which it is analyzed. This work also clearly shows that modern molecular diagnostics are used not only to classify or confirm a diagnosis but also to feed into predictive tools, with a concrete impact on follow-up and treatment decisions.

A second crucial strand of the Research Topic concerns the development of more refined diagnostic and prognostic tools for risk stratification. From this perspective, the work of Golding et al. is of particular interest because it proposes mRNA-expression-based classifiers that can predict, in the preoperative phase, a low risk of lymph node invasion and metastasis in thyroid cancers. This is an important conceptual step: not to limit oneself to detecting mutations or fusions, but to use the transcriptome as a surrogate for the tumor’s active biological state. This approach appears particularly promising for reducing overtreatment and guiding more conservative decisions in patients with low-risk lesions, especially in the gray area of Bethesda III-VI nodules. The work also underscores the potential of combining RNA sequencing and machine learning, showing that “next-generation diagnostics” does not simply coincide with high-throughput sequencing but with the ability to transform large volumes of biological data into clinically usable information.

At a similar but more biology-oriented level, the review by Harvey et al. further expands the discussion, arguing that the transcriptome represents an interpretative level closer to the real clinical behavior of papillary thyroid carcinoma than the genotype alone does. The authors show that transcriptomic subtypes, differentiation programs, interaction with the microenvironment, and sensitivity to radioactive iodine can be captured more accurately by gene expression signatures than mutational drivers alone. The contribution is particularly relevant in the current scenario, in which overdiagnosis and the risk of overtreatment make a biologically informed approach to decisions on active surveillance, surgical extension, radiometabolic therapy, and follow-up intensity increasingly necessary. This article provides, in essence, the theoretical framework within which to read many of the other works of the Research Topic: the mutation identifies the possibility, the transcriptome describes the functional state.

A third axis concerns improving the preoperative differential diagnosis of follicular neoplasms, a historically problematic topic in thyroid cytology. The study by Zhu et al. addresses this problem by proposing an innovative parameter, the thyroglobulin-to-tumor volume ratio (Tg/Vol), which is integrated with contrastographic ultrasound features to distinguish follicular adenoma from follicular carcinoma. Although subject to the intrinsic limitations of a single-center retrospective study, the work is interesting because it reintroduces the value of a classical biomarker, thyroglobulin, in a quantitative manner, correcting for tumor size and incorporating it into a combined model. In an era dominated by sequencing, this contribution is a fitting reminder that precision medicine does not necessarily coincide with pure genomic analysis, but with the intelligent integration of biochemical biomarkers, advanced imaging, and statistical modeling.

Still in the field of biomarkers and risk factors, Lin et al. explored the relationships among thyroid nodules, iodine nutritional status, thyroglobulin, and other factors in a 1:1 case-control study. Although the work is not strictly focused on genomics, its inclusion in the Research Topic is pertinent because it highlights the importance of environmental and metabolic context in thyroid biology. In particular, the association of high levels of Tg and TgAb with the presence of nodules suggests that a modern characterization of thyroid lesions cannot disregard the interplay among molecular profiles, thyroid function, autoimmunity, and nutritional factors. The contribution, therefore, broadens the Research Topic’s perspective beyond the manifest tumor towards the complexity of the biological terrain on which it develops.

The Research Topic also includes a more exploratory and experimental contribution by Yu et al., which identifies *E2F1* as a gene associated with cellular senescence and prognosis in papillary thyroid carcinoma, combining bioinformatic analyses of public datasets with *in vitro* experimental validation. Perhaps the most interesting finding is the apparent association between high *E2F1* expression and a more favorable prognosis, in contrast to the role classically attributed to E2F1 in other oncological contexts. Beyond the necessary interpretative cautions, the work shows how integrating bioinformatics, transcriptomics, and cell biology can identify new biomarker candidates and pathogenetic hypotheses, especially in the context of the relationship among proliferation, senescence, the immune microenvironment, and tumor progression.

Finally, the case report by Gualandi et al. on a *DICER1*-Wild-Type thyroblastoma represents perhaps the rarest and most conceptually stimulating contribution of the Research Topic. Through long-read Oxford Nanopore sequencing and integrated pathway analysis, the authors describe an alternative oncogenic structure characterized by AGK-BRAF fusion, *EIF1AX* duplication, and *TERT* promoter mutations. This work is paradigmatic of the value of next-generation techniques in ultra-rare tumors: not only to identify unexpected alterations, but to redefine the molecular boundaries of pathological entities. In this case, the absence of canonical *DICER1* mutations does not rule out the diagnosis but suggests a biologically distinct subgroup with diagnostic and potentially therapeutic implications. The paper eloquently demonstrates how long-read sequencing and integrated bioinformatics analysis can play a decisive role even outside the most frequent tumors.

Taken together, the nine contributions in this volume outline some key messages. First, the molecular characterization of thyroid lesions has moved beyond the simple identification of canonical drivers and is now advancing towards integrated models that combine DNA, RNA, pathway biology, clinical data, and predictive tools. Second, the notion of precision medicine in thyroid pathology does not coincide with the mere possibility of prescribing a targeted therapy, but rather encompasses the ability to select whom to observe, whom to operate on, how long to extend surgery, whom to treat with radioactive iodine, and how to modulate follow-up. Third, “next generation techniques” are not just sequencing platforms, but a real conceptual infrastructure that includes machine learning, transcriptomic analysis, prognostic models, multi-omics integration, and biological interpretation of variants.

Of course, the challenges of prospective validation, methodological harmonization, and the transferability of these tools in daily clinical practice remain open. Many of the papers collected in the Research Topic are retrospective, monocentric, or exploratory, and will require confirmation in independent cohorts and multicenter studies. However, the value of this volume lies precisely in its collection of contributions that, from different yet converging perspectives, show how thyroid disease is entering a new phase: one in which the molecular profile is no longer ancillary to diagnosis but an integral part of the disease’s biological definition.

In conclusion, this Research Topic offers an up-to-date and stimulating overview of the most promising directions of contemporary thyroid research. From the genomics of rare tumors to prognostic transcriptomics, from serological biomarkers to clinical-molecular predictive models, the contributions collected here confirm that molecular diagnostics is no longer an optional complement but a central hub for understanding and managing thyroid lesions. This volume clearly demonstrates that the future of the thyroid will not be defined by a single mutation but by the ability to read the tumor as a complex, clinically interpretable biological system.

